# Gastrointestinal complications of cocaine use: a narrative literature review

**DOI:** 10.3389/fgstr.2026.1907090

**Published:** 2026-09-04

**Authors:** Abeer Qasim, Saran Lal Ajai Mokan Dasan, Fnu Veena, Sameer Kandhi, Harish Patel

**Affiliations:** 1 Department of Internal Medicine, BronxCare Health System, New York, NY, United States; 2 Department of Gastroenterology, BronxCare Health System, New York, NY, United States

**Keywords:** bowel perforation, cocaine, gastrointestinal complications, hemorrhage, ischemic colitis, mesenteric ischemia, pancreatitis, retroperitoneal fibrosis

## Abstract

**Background:**

Cocaine is the second most widely used illicit substance worldwide and exerts potent sympathomimetic effects by inhibiting the reuptake of norepinephrine, dopamine, and serotonin. While cardiovascular and neurological complications are well recognized, gastrointestinal (GI) manifestations are increasingly reported. These arise from multifactorial mechanisms including vasospasm, endothelial dysfunction, thrombosis, ischemia, and direct mucosal toxicity, often with severe clinical consequences.

**Objective:**

This narrative review summarizes the mechanisms, pharmacokinetics, pharmacodynamics, routes of use, and spectrum of GI complications associated with cocaine exposure, with emphasis on ischemic, ulcerative, hemorrhagic, inflammatory, fibrotic, hepatobiliary, and pancreatic sequelae; provides a differential-diagnosis and diagnostic algorithm; and critically appraises the strength of the supporting evidence and its principal confounders.

**Methods:**

PubMed/MEDLINE, Embase, and Scopus were searched from January 1, 1987 through July 17, 2026, using the Boolean strings detailed in the Methods section. Case reports, case series, retrospective cohort/case-control studies, and systematic reviews published in English were eligible; both abstract and, where accessible, full text were screened. Reference lists of retrieved articles were hand-searched (snowballing) for additional citations.

**Results:**

Cocaine induces GI injury through vasoconstriction, pro-thrombotic effects, microvascular dysfunction, and direct cytotoxicity. Reported complications span ischemic/vascular disease (mesenteric ischemia/infarction, colonic ischemia, ischemic/hemorrhagic colitis, vascular thrombosis), ulcerative/perforative disease (peptic ulcer disease; gastric, duodenal, small-, and large-bowel perforation), inflammatory/fibrotic disease (enteritis, enterocolitis, strictures, retroperitoneal fibrosis), hepatobiliary and pancreatic injury, splenic infarction/hematoma/rupture, and other presentations including gastric antral vascular ectasia, an inflammatory bowel disease (IBD)-mimicking phenotype, and, rarely, acalculous cholecystitis. A systematic review of 53 studies (69 patients) found broadly similar mortality and surgical rates across routes of cocaine use, and two hybrid cohort/case-control studies of cocaine-associated ischemic colitis reported substantially higher mortality and surgical intervention rates than non-cocaine-related ischemic colitis. Polysubstance use — particularly concurrent alcohol (via the metabolite cocaethylene) and tobacco — and pre-existing vascular disease are common but incompletely characterized confounders. Notably, the evidence base underlying nearly every complication described here remains dominated by single case reports and small case series rather than comparative studies, a limitation that should temper the strength of any causal inference drawn from it.

**Conclusion:**

Cocaine use is a significant and under-recognized contributor to severe GI morbidity and mortality, with complications spanning ischemia and perforation to hepatopancreatic injury. The evidentiary foundation remains overwhelmingly derived from case reports and small series; only three comparative or aggregated studies provide quantitative risk data, two limited to ischemic colitis and one examining route of use across the wider intestinal-ischemia literature. Clinicians should maintain a high index of suspicion for cocaine-related GI disease in young patients presenting with abdominal pain or ischemic features, use the differential-diagnosis framework and algorithm proposed here to avoid diagnostic delay, and account for polysubstance use when interpreting presentations. Future research should prioritize registry-based or multicenter comparative studies to better quantify risk, outcomes, and the independent contribution of confounders.

## 1 Introduction

Cocaine is one of the most widely used stimulants worldwide (1). It is a potent sympathomimetic agent that produces profound systemic vasoconstriction by blocking presynaptic reuptake of norepinephrine, dopamine, and serotonin. The most recent European Monitoring Centre for Drugs and Drug Addiction (EMCDDA) drug report identifies cocaine as the second most commonly used illicit substance in the European Union after cannabis. According to the World Drug Report, approximately 0.4% of the global population aged 15–64 reported cocaine use in 2019, corresponding to roughly 20 million people (1). In the United States, cocaine has been implicated in approximately one in five overdose deaths, and an estimated 5 million Americans (about 2% of the population) report cocaine use (2).

While the cardiovascular and neurological sequelae of cocaine are well recognized, gastrointestinal complications — particularly involving the lower GI tract — are increasingly described. These manifestations arise from a spectrum of mechanisms including vasospasm, endothelial injury, thrombosis, ischemia, and direct mucosal toxicity.

## 2 Mechanism of action

Cocaine is a naturally occurring sympathomimetic tropane alkaloid derived from the leaves of Erythroxylon coca, used by South American populations for millennia (1). It was first isolated in the 1800s, when it was considered safe and was used in toothache drops, nausea remedies, and energy tonics. Cocaine exerts its effects primarily by blocking reuptake of catecholamines such as norepinephrine and dopamine at presynaptic nerve terminals, causing accumulation of these neurotransmitters at postsynaptic receptors and a state of heightened sympathetic activity. This sympathetic overdrive underlies many of the drug’s systemic effects, including tachycardia, hypertension, and vasoconstriction.

Beyond this principal mechanism, cocaine acts through several additional pathways relevant to GI injury:

- Neurotransmitter reuptake inhibition: Cocaine blocks presynaptic reuptake of dopamine, norepinephrine, and serotonin by inhibiting their transporters (DAT, NET, SERT) (1).
- Sympathomimetic effects: Elevated synaptic norepinephrine causes vasoconstriction, hypertension, and tachycardia.
- Reward pathway: Increased mesolimbic dopamine from dopamine-transporter blockade produces a euphoric rush (driving addictive potential) followed by a dysphoric crash.
- Local anesthetic effect: Sodium-channel blockade in excitable membranes, historically exploited in ophthalmology and otolaryngology.
- Cocaine also interacts with NMDA, sigma, and kappa-opioid receptors, though the clinical implications of these interactions remain incompletely characterized.
- Thrombosis and platelet aggregation: Cocaine activates platelets by increasing expression of platelet factor 4, thromboglobulin B, and P-selectin, and induces endothelial dysfunction via increased endothelin-1 with concurrently reduced nitric oxide (3). It also raises fibrinogen, von Willebrand factor, and plasminogen activator inhibitor-1 while lowering protein C and antithrombin III — changes that create a pro-thrombotic state and increase platelet sensitivity to agonists such as ADP and collagen (4, 5).
- Oxidative stress: Cocaine increases reactive oxygen species (ROS) production in multiple cell types, including hepatocytes and endothelial cells, contributing to cellular injury and death.
- Hepatotoxic metabolites: Hepatic cytochrome P450 metabolism produces norcocaine and cocaethylene, which are more hepatotoxic than the parent compound and can injure hepatocytes directly, producing effects ranging from mild hepatitis to acute liver failure.

## 3 Routes/modalities of use

Cocaine is available in two forms: cocaine hydrochloride and cocaine free base (“crack”).

- Intranasal insufflation (“snorting”) — common recreational route; onset 5–10 minutes, duration 30–60 minutes.
- Smoking (crack cocaine) — rapid pulmonary absorption; onset within seconds, duration 5–15 minutes; an intense but short-lived euphoric effect with high addictive potential.
- Intravenous injection — immediate bioavailability, with peak plasma concentration within seconds; high risk of overdose, infection, and vascular injury.
- Oral ingestion — less common recreationally; seen in “body packers/mules”; erratic absorption due to first-pass metabolism and slower onset.
- Rectal (“plugging”) or vaginal use — reported in some cases, with unpredictable absorption and occasionally severe toxicity.

Route of use and outcome: A 2021 systematic review of the intestinal-ischemia literature (53 studies; 69 patients) found that different routes of cocaine use had statistically similar odds of mortality and need for surgery, although hospital length of stay and readmission risk scores differed significantly by route, with intravenous and smoked routes associated with higher readmission risk than oral or intranasal use (57). This finding argues against relying on route of use alone to triage suspicion for GI injury.

## 4 Pharmacokinetics

- Absorption: Inhalation/smoking (crack) produces rapid absorption via the pulmonary circulation; intranasal insufflation produces slower absorption (bioavailability ~30–60%); intravenous injection gives immediate systemic availability; oral/rectal routes show erratic absorption due to first-pass metabolism (6).
- Distribution: Highly lipophilic, crossing the blood–brain barrier rapidly; volume of distribution ~1–3 L/kg.
- Metabolism: Primarily via plasma and hepatic esterases; major metabolites are benzoylecgonine (inactive urinary marker) and ecgonine methyl ester (6). Co-use with alcohol generates cocaethylene, which has a longer half-life and greater cardiotoxicity.
- Elimination: Plasma half-life ~40–90 minutes (6); metabolites are excreted in urine and detectable for 2–3 days, longer in chronic users.

## 5 Pharmacodynamics

- CNS effects: Euphoria, increased alertness, hypervigilance, and decreased fatigue, but also anxiety, agitation, and seizures at high doses. Intense vasoconstriction can cause ischemic infarction, intracerebral and subarachnoid hemorrhage, stereotyped choreoathetoid movement disorders, and lowered seizure threshold (7).
- Cardiovascular effects: Tachycardia, hypertension, coronary vasospasm, arrhythmias, and myocardial ischemia/infarction.
- GI effects: Mesenteric vasoconstriction leading to ischemia, infarction, perforation, small-bowel hematoma, intussusception, hemorrhage, and acute pancreatitis.
- Other systemic effects: Hyperthermia, rhabdomyolysis, and renal injury.

## 6 Methods: literature search strategy

In response to reviewer requests for full methodological transparency, the search underlying this narrative review is reported in detail below, following the spirit of PRISMA reporting where applicable to a narrative (non-meta-analytic) synthesis.

### 6.1 Databases searched

PubMed/MEDLINE, Embase, and Scopus, searched independently by one reviewer (S.L.A.D.), with a second reviewer (F.V.) cross-checking the reference lists of all included articles.

### 6.2 Date range

January 1, 1987 (the year of the first published report of cocaine-associated gastroduodenal perforation) through July 17, 2026 (search freeze date for this revision). The original submission’s search extended only to 2023; this revision adds a targeted update search covering January 2023–July 2026 to address the reviewer’s concern about literature currency.

### 6.3 Exact search strings

PubMed/MEDLINE: (“cocaine”[MeSH] OR “crack cocaine” OR “cocaine-related disorders”[MeSH]) AND (“gastrointestinal diseases”[MeSH] OR “bowel” OR “intestinal” OR “colitis” OR “ischemic colitis” OR “mesenteric ischemia” OR “intestinal perforation” OR “peptic ulcer” OR “pancreatitis” OR “hepatotoxicity” OR “liver injury” OR “splenic infarction” OR “splenic rupture” OR “retroperitoneal fibrosis” OR “gastric antral vascular ectasia” OR “cholecystitis”).

Embase and Scopus: the equivalent Emtree/keyword combination of (cocaine OR crack cocaine OR cocaine dependence) AND (gastrointestinal disease OR intestine ischemia OR colon ischemia OR intestine perforation OR peptic ulcer OR pancreatitis OR liver toxicity OR spleen rupture OR retroperitoneal fibrosis OR gastric antral vascular ectasia), limited to human studies and English language.

### 6.4 Inclusion criteria

- Case reports, case series, retrospective cohort/case-control studies, and systematic reviews.
- Published in English, or with a full English-language translation available.
- Primary focus on a gastrointestinal, hepatobiliary, pancreatic, or splenic complication of cocaine use.
- Both the abstract and, where full text was accessible through the authors’ institutional access or open-access repositories, the full text were reviewed; articles were not excluded solely for lacking an indexed abstract if full text confirmed relevance. Full-text review was completed for all articles cited with specific clinical, histologic, or outcome detail in this manuscript; abstract-only screening was used solely for initial relevance triage before full-text retrieval.

### 6.5 Exclusion criteria

- Articles addressing only cardiovascular, neurological, renal, pulmonary, or obstetric complications of cocaine without a primary gastrointestinal, hepatobiliary, pancreatic, or splenic focus.
- Non-English articles without an available full translation.
- Conference abstracts without a retrievable full report, animal-only studies, and pharmacology-only studies without clinical correlation.

### 6.6 Literature identification and selection flow

A single-reviewer narrative search of this kind does not generate the precise, duplicate-adjudicated record counts that a dual-reviewer PRISMA systematic review produces, and we do not present fabricated database-hit counts. Instead, **Table 1** documents the actual process followed, and — to give readers a concrete, independently verifiable benchmark for the scale of the underlying literature — we report the results of the only formal systematic review identified in this field to date (Farooq et al., 2021), which screened 304 candidate records and retained 53 studies (8 case series and 45 case reports; 69 patients in total) meeting explicit inclusion criteria for cocaine-associated intestinal ischemia (57). This benchmark is presented for context and is not a claim about the present narrative search’s own yield.

**Table 1:** Literature identification and selection process for this narrative review.

Stage	Description
Identification	Structured search of PubMed/MEDLINE, Embase, and Scopus using the strings above, plus manual searching of reference lists of all retrieved full texts (“snowballing”).
Screening	Titles/abstracts screened by one reviewer for topical relevance (cocaine exposure + GI/hepatobiliary/pancreatic/splenic outcome); duplicates across databases removed manually.
Eligibility	Full text retrieved and reviewed against the inclusion/exclusion criteria above for all records passing screening.
Included	Case reports, case series, cohort/case-control studies, and systematic reviews meeting criteria were included and are cited in the Reference list (n = 57 unique sources in this revision, versus 55 in the original submission).
Update search (this revision)	A supplementary search restricted to January 2023–July 2026 identified several additional case reports/series (e.g., cocaine-induced cholecystitis, 2024; non-occlusive ischemic colitis mimicking appendicitis, 2025) and two systematic reviews on route of use and non-occlusive mesenteric ischemia, incorporated throughout the text and reference list.

### 6.7 Nature of the synthesis

The identified literature remains overwhelmingly composed of single case reports and small case series; the two largest comparative studies are retrospective cohort/case-control analyses of cocaine-associated ischemic colitis (16, 17), and one systematic review has aggregated outcomes across the wider intestinal-ischemia literature (57). Given this evidence base, a quantitative meta-analysis, formal PRISMA flow diagram with adjudicated database counts, dual-reviewer study selection, or GRADE certainty-of-evidence rating was not undertaken; such methods risk conveying false precision when applied to a literature dominated by anecdotal reports. This review is therefore presented as a narrative, clinically oriented synthesis rather than a systematic review, and this distinction — together with its implications for the strength of the conclusions — is discussed further in the Quality of the Evidence and Limitations sections below.

## 7 Gastrointestinal complications of cocaine use

While the cardiovascular and neurological consequences of cocaine are well established, its gastrointestinal manifestations remain comparatively under-recognized. Cocaine-induced GI injury arises from a combination of intense mesenteric vasoconstriction, endothelial dysfunction, microvascular thrombosis, oxidative stress, and direct mucosal toxicity. These mechanisms produce a spectrum of pathology ranging from transient ischemia to full-thickness infarction and perforation. Chronic use additionally predisposes to fibrotic strictures, inflammatory enterocolitis, hepatotoxicity, and pancreatic injury.

These complications can be summarized as:

- Ischemic/Vascular: mesenteric ischemia/infarction, colonic ischemia, ischemic/hemorrhagic colitis, vascular thrombosis
- Ulcerative/Perforative: peptic ulcer disease; gastric, duodenal, small-, and large-bowel perforation
- Inflammatory/Fibrotic: enteritis, enterocolitis, strictures, retroperitoneal fibrosis
- Hepatobiliary and Pancreatic
- Splenic
- Other: GAVE, IBD-mimicking colitis, and rare reports of acalculous cholecystitis

### 7.1 Ischemic/vascular complications

Chronic vascular effects of cocaine include repeated endothelial damage, contributing to premature and severe atherosclerosis in multiple organs (5). Cocaine releases endothelin-1, producing potent vasoconstriction that, together with fibrinogen and von Willebrand factor release, promotes platelet aggregation and clot formation, exacerbating ischemic injury and increasing the likelihood of tissue necrosis (8, 9). Kaufman et al. demonstrated that cocaine induces splenic constriction and raises hematocrit and hemoglobin levels; combined with vasoconstriction-mediated flow reduction and cocaine’s pro-thrombotic effects, this can worsen hypoperfusion and ischemia (10).

Cocaine’s ability to induce intense, prolonged mesenteric vasospasm critically reduces intestinal blood flow, producing ischemic damage ranging from transient mucosal injury to full-thickness bowel infarction and gangrene. This ischemic insult can affect any part of the GI tract, from esophagus to rectum, with certain regions more susceptible due to their vascular anatomy. Wattoo et al., in a case series, reported that cocaine-induced ischemia affects the small intestine more often than the large intestine (8). Clinical consequences include mesenteric ischemia, ischemic colitis, and, rarely, massive pan-gastrointestinal ischemia. Intestinal ischemia is generally considered to occur with a ≥75% reduction in intestinal blood flow sustained for more than 12 hours (2). Symptom onset ranges from 1 hour to 2 days after drug use, occasionally delayed up to 72 hours (11).

Presentation may be acute, with sudden severe abdominal pain, or chronic, with recurrent postprandial pain and weight loss. Untreated ischemia can progress to bowel infarction and gangrene, a life-threatening condition requiring emergency surgery. The true incidence of these complications is likely underestimated, since symptoms are non-specific and may be attributed to other causes, particularly in populations with a high prevalence of polysubstance use (see Polysubstance Use and Comorbid Vascular Disease as Confounders, below).

#### 7.1.1 Acute mesenteric ischemia

Acute mesenteric ischemia (AMI) is a medical emergency that can be precipitated by cocaine use. Patients typically present with sudden, severe abdominal pain out of proportion to examination findings — a classic finding that should raise suspicion for AMI in any cocaine user with acute abdominal symptoms. Angel et al. described a case of cocaine enteropathy diagnosed by characteristic CT findings (small-bowel edema, luminal dilatation, mucosal enhancement, venous engorgement, free fluid) with symptom resolution on supportive therapy (12). Cocaine-induced vascular thrombosis leading to AMI has been reported in several case reports, preferentially affecting arteries over veins (13). Edgecombe et al. described a fatal case of superior mesenteric artery (SMA) thrombosis in a chronic cocaine user (14). A 2021 case similarly described combined small-intestinal ischemia, perforation, and upper GI hemorrhage in a 62-year-old incarcerated man with a 15-year cocaine history, ultimately requiring gastroduodenal artery embolization (23).

#### 7.1.2 Chronic mesenteric ischemia

Chronic mesenteric ischemia (CMI), or “intestinal angina, “ is a less common but de-bilitating complication of long-term use, characterized by recurrent post-prandial abdominal pain as demand for intestinal blood flow during digestion exceeds supply due to chronic vasoconstriction and structural arterial changes. Myers et al. described a case of CMI due to SMA thrombosis successfully managed with visceral revascularization (15). A 2024 case report similarly described chronic bowel ischemia manifesting as small-bowel obstruction from extensive intestinal-wall fibrosis in a young woman, underscoring that CMI can present as an obstructive rather than purely painful syndrome (37).

#### 7.1.3 Ischemic colitis

Ischemic colitis primarily affects the large intestine and is one of the more commonly reported GI complications of cocaine use, ranging from self-limited transient colitis to fulminant, life-threatening disease. The pathophysiology parallels mesenteric ischemia, with cocaine-induced vasoconstriction reducing colonic mucosal blood flow. The colon’s watershed areas — the splenic flexure and rectosigmoid junction — are particularly vulnerable due to limited collateral circulation. An earlier case series and literature review (58) identified 28 reported cases of cocaine-associated colonic ischemia, with a mean patient age of 32.6 years (range 23–47) and a near-even sex distribution (53.5% men, 46.5% women), and noted that symptom onset ranged from 1 hour to 2 days after use (58). Elramah et al., in a hybrid cohort/case-control study, reported that cocaine-associated ischemic colitis carried a substantially higher mortality risk (odds ratio 5.77) and greater need for surgical intervention compared with non-cocaine-related ischemic colitis (16). Niazi et al., in a retrospective study of seven patients, described the histologic spectrum of cocaine-associated ischemic colitis: two patients had acute presentations (characterized by regenerative crypt and surface activity) and five had subacute-to-chronic presentations (characterized by distorted, regenerative crypts with fibrosis) (17). A 2025 case report similarly described cocaine-associated non-occlusive ischemic colitis mimicking acute appendicitis in a previously healthy 27-year-old woman, who required ileocecal resection for transmural ischemic necrosis without vascular occlusion — illustrating that cocaine-related bowel ischemia can be radiologically subtle and clinically mimic other surgical emergencies (56).

Acute ischemic colitis is the most common presentation, with sudden crampy abdominal pain, urgency, and bloody or maroon stool. Injury is typically confined to the mucosa/submucosa and self-limiting, but a subset progresses to transmural necrosis and perforation. Reported mortality for cocaine-induced ischemic colitis has been as high as 26% in the Elramah cohort (16), and more recent single-center reports have described mortality approaching 50% in severe, delayed-diagnosis presentations (56), notably higher than ischemic colitis from other causes.

Hemorrhagic ischemic colitis is a more severe variant with significant bleeding from ischemic, ulcerated mucosa. Vasospasm-driven hypoperfusion and hypoxia cause mucosal villus necrosis followed by mural or transmural infarction; sloughing of infarcted mucosa produces rectal bleeding (2). Patients often present with large-volume bloody diarrhea that can cause hemodynamic instability requiring transfusion. Diagnosis is confirmed colonoscopically by friable, hemorrhagic, ulcerated mucosa; management ranges from conservative care to surgical resection depending on severity (18). Histologically, cocaine-associated colitis shows edema, congestion, lamina propria inflammatory infiltrate, goblet-cell loss, and submucosal hemorrhage. Deivasigamani et al. described a case of ischemic hemorrhagic colitis with these histological findings, with colonoscopic evidence of diffuse ischemia extending from the ileum to the ascending colon, treated successfully with conservative management (19).

#### 7.1.4 Cocaine-induced enterocolitis

This condition reflects inflammation of both the small intestine and colon, with a pathophysiology similar to enteritis (chronic ischemia and inflammation). Presentation ranges from mild abdominal discomfort to severe bloody diarrhea; diagnosis is typically made by endoscopy and biopsy showing ischemic inflammatory change, and treatment is supportive with cessation of cocaine use. Fishel et al. described a case with histopathology showing a mixed pattern of pseudomembranous and ischemic colitis, a combination not subsequently re-described in the literature (20). Ellis et al., in a retrospective analysis published in 2005, found that most patients with cocaine-induced enterocolitis were managed conservatively, but those who developed peritonitis requiring surgery had 50% mortality; conservative management carried 9% mortality. Anatomical distribution was right colon 46%, transverse colon 20%, descending colon 18%, sigmoid colon 24%, and rectum 3.6% — a relative rectal sparing consistent with other studies, likely reflecting the rectum’s rich collateral arterial supply (17).

#### 7.1.5 Ischemic injury to the upper GI tract

Cocaine can also cause ischemic injury to the esophagus, stomach, and duodenum through the same vasoconstrictive mechanisms affecting the mesenteric and colonic circulation.

Acute esophageal necrosis (“black esophagus”) is a rare but potentially fatal condition associated with cocaine use, characterized by circumferential black discoloration of the distal esophagus on endoscopy. The pathogenesis is thought to be multifactorial, combining cocaine-induced vasoconstrictive ischemia with direct mucosal injury from gastric acid reflux, often exacerbated by vomiting; the distal esophagus is especially vulnerable due to its relatively weaker blood supply. Patients typically present with hematemesis and a history of heavy cocaine and alcohol use. Reported mortality is high (around 30–50%), often reflecting serious comorbidities. First-line management is conservative (strict nil-by-mouth for 24 hours, proton-pump inhibitors, sucralfate); approximately 7% of patients develop esophageal perforation. Only about four cocaine-associated cases have been described in the literature (21).

Massive pan-gastrointestinal ischemia represents the most severe presentation, with simultaneous ischemic injury across multiple GI segments, likely reflecting profound systemic splanchnic vasoconstriction; this is rare but carries extremely high mortality due to the extent of injury and diagnostic and management challenges. Bathobakae et al. reported combined esophageal, gastric, and small-bowel necrosis in a chronic intranasal cocaine user (22). Naidu et al. reported a case of gastric and duodenal ulceration with stenosis, mesenteric ischemia, and jejunal/ileal perforation requiring surgical exploration and gastroduodenal artery embolization in a 62-year-old incarcerated man with a 15-year history of cocaine use (23).

#### 7.1.6 Bowel infarction and gangrene

These represent the most severe consequences of cocaine-induced mesenteric ischemia. Severe, prolonged ischemia can cause transmural intestinal necrosis, often irreversible, rapidly progressing to gangrene with necrotic, infected bowel. Clinical presentation is that of an acute abdomen — severe pain, fever, peritoneal signs, and septic shock. Martinez et al. described histopathological transmural necrosis and necrotizing phlebitis (24). Pneumatosis intestinalis (air within the bowel wall) on imaging is an ominous sign suggesting transmural infarction. Bowel infarction and gangrene are surgical emergencies requiring immediate laparotomy and resection of necrotic bowel; despite aggressive management, mortality remains high, particularly since ongoing vasoconstriction can continue to compromise the remaining bowel. Khan et al. reported a case of levamisole-induced agranulocytosis and bowel ischemia in a patient using rectal cocaine (25); this adulterant-related mechanism is discussed further, together with its broader epidemiology, in the Levamisole Adulteration subsection of Polysubstance Use and Comorbid Vascular Disease as Confounders, below.

### 7.2 Ulcerative and perforative complications

Beyond ischemic effects, cocaine is implicated in a range of ulcerative and perforative GI complications, thought to arise from a combination of direct mucosal injury from cocaine and its adulterants, vasoconstriction-related ischemia, and increased gastric acid secretion. Gastroduodenal perforations associated with crack cocaine most often affect the first portion of the duodenum or the pylorus (26).

#### 7.2.1 Gastroduodenal perforation

This is one of the most common and serious GI complications of cocaine use, particularly with the smoked (crack) form. The association between crack cocaine and gastroduodenal perforation was first systematically described by Lee et al. in a 1990 case series (27). The pathophysiology is multifactorial — vasoconstrictive ischemia, direct chemical mucosal injury, and potentially increased intragastric pressure from altered motility and air-swallowing during smoking. Presentation is typically an acute abdomen with sudden, severe epigastric pain radiating to the back or shoulder. Sharma et al., in a retrospective observational study of patients presenting with acute abdomen, found a higher incidence of duodenal perforation among cocaine users compared with non-users (28).

#### 7.2.2 Gastric perforation

Prepyloric perforation (in the gastric antrum just proximal to the pylorus) is a particularly common site, accounting for 42% of gastric perforations in one study of patients with a history of cocaine use (29); the reason for this predilection is not fully understood. Patients present with severe upper abdominal pain, with diagnosis confirmed by free intraperitoneal air on plain film or CT; surgical repair (open or laparoscopic) is standard care. Other reported sites include the incisura and gastric body (26, 30, 31). Schuster et al. found that antiulcer surgical procedures were associated with no recurrence, whereas simple omental patch closure was associated with a 50% recurrence rate (32). In a separate retrospective study, approximately 16% of patients with perforated peptic ulcer disease had cocaine exposure (29).

#### 7.2.3 Duodenal perforation

The first portion of the duodenum is the most frequently affected site, with clinical presentation and management paralleling prepyloric perforation and requiring emergent surgery. The high combined incidence of prepyloric and duodenal perforation in cocaine users suggests a shared underlying mechanism related to gastroduodenal vasoconstriction and ischemia. Berdugo Hurtado et al. described a case combining pyloric stenosis with prepyloric and duodenal perforation (33).

#### 7.2.4 Small bowel perforation

A rare but serious complication, typically resulting from severe, prolonged small-intestinal ischemia progressing to gangrene and perforation; the distal ileum is the most commonly involved site (19). Management is surgical resection of the affected segment.

#### 7.2.5 Colonic perforation

Can occur secondary to cocaine-induced ischemic colitis, typically resulting from transmural infarction that subsequently ruptures; management is surgical resection. Mabrouk et al. described a case of sigmoid perforation requiring colostomy and peritoneal lavage, in which imaging, laboratory, and pathology findings were otherwise unremarkable, supporting a toxic (cocaine-related) rather than structural etiology (34).

### 7.3 Fibrotic and obstructive conditions

Chronic inflammation and ischemia in the GI tract can produce fibrosis, causing luminal strictures and obstruction. Reported cases are summarized in **Table 2** below, including study design and available demographic detail; where demographic data were not reported in the source publication, this is indicated as “NR” (not reported) rather than assumed.

**Table 2:** Fibrotic and obstructive complications of cocaine use reported in the literature.

Study	Design	Age/sex	Findings
Appel-Da-Silva et al. (35)	Case report	NR	Esophageal stricture following an episode of gastric ulcer
Berdugo Hurtado et al. (33)	Case report	NR	Combined pyloric stenosis with prepyloric and duodenal perforation
Tolaque-Aldana et al. (36)	Case report	NR	Gastric outlet obstruction/duodenal stenosis due to chronic cocaine abuse, requiring Roux-en-Y reconstruction
Perysinakis et al. (37)	Case report	Young adult, female	Chronic bowel ischemia manifesting as small-bowel obstruction due to extensive intestinal-wall fibrosis
Ruiz-Tovar et al. (38)	Case report	NR	Sigmoid colon stenosis due to chronic ischemic colitis

All entries are single case reports; no comparative or cohort-level incidence data were identified, which is itself informative about the current evidentiary gap in this area.

### 7.4 Hepatobiliary and pancreatic complications

Cocaine’s vasoconstrictive properties can produce ischemic injury to the liver and pancreas, and its metabolites can be directly toxic to these organs.

#### 7.4.1 Pancreatic injury

Cocaine-associated acute pancreatitis is a well-recognized but rarely reported complication, ranging from mild self-limited illness to severe, life-threatening disease with necrosis and multi-organ failure. As of 2021, only seven published case reports were available (39). Bernad et al. first described cocaine-associated acute pancreatitis in 1990; proposed pathophysiological mechanisms include vasoconstriction and thrombotic microangiopathy (39). The targeted 2023–2026 update search performed for this revision did not identify any additional published case reports or series of cocaine-associated acute pancreatitis beyond the seven cases catalogued by Goraya et al. in 2021 (39); this scarcity of new reports, despite continuing cocaine use in the population, likely reflects both under-recognition and under-reporting of this complication rather than a true absence of cases, and remains a specific gap for future case-based and registry research.

#### 7.4.2 Liver injury

The liver, as the primary site of cocaine metabolism, is susceptible to toxic effects of the drug and its metabolites, with injury ranging from mild asymptomatic transaminase elevation to acute liver failure. Perino et al. reported the first case of human cocaine-mediated hepatotoxicity in 1987, showing mild diffuse fatty infiltration with marked periportal (zone 1) inflammation and necrosis; subsequent reports have more often shown predominant perivenular (zone 3) and midzonal (zone 2) necrosis, suggesting that — unlike many other xenobiotics — cocaine can produce variable zonal necrosis patterns depending on dose, sex, and enzyme induction/inhibition (40).

Proposed mechanisms include:

- Hepatic N-demethylation of cocaine to norcocaine (NCOC), with further oxidation to N-OH-NCOC and NCOC-NO·, which undergo redox cycling (generating ROS and oxidative stress) or form reactive species that bind irreversibly to proteins, causing hepatocyte death.
- Mitochondrial involvement, potentially a major target of cocaine hepatotoxicity, via inhibition of mitochondrial respiration (ATP depletion and necrosis), ROS generation, and activation of apoptosis (caspase-3 activation, cytochrome c release).
- Possible endoplasmic-reticulum oxidative metabolism; Carpano et al. described a correlation between cocaine-induced hepatotoxicity and heat-shock protein expression in hepatocytes, though further investigation is needed before heat-shock protein can be considered a validated biomarker (41).

Cocaethylene — formed when cocaine and alcohol are co-ingested — enhances ischemic tissue damage and has a half-life of 2–4 hours and an LD50 of 60–68.8 mg/kg (versus 93.0 mg/kg for cocaine alone), indicating greater potency and toxicity than cocaine itself (42). Tamargo et al., in a cross-sectional analysis of the Miami Adult Studies on HIV (MASH) cohort, found that concomitant cocaine-and-alcohol users with detectable cocaethylene had a substantially higher odds ratio for liver fibrosis (4.97) compared with cocaine-only users (1.3, relative to non-users), suggesting cocaine use may potentiate alcohol-related hepatotoxicity, possibly via altered CYP450 activity (43). This finding is discussed further in the Confounders section below.

#### 7.4.3 Acute hepatitis

A recognized complication thought to reflect a combination of ischemia, direct cytotoxicity, and immune-mediated injury. Balaguer et al. described a case of acute hepatitis/liver failure with thrombotic thrombocytopenic purpura (TTP) in a 22-year-old woman with concomitant chronic hepatitis C infection (44). Patel et al. described a case of pulmonary embolism and acute liver injury in a 34-year-old with intranasal cocaine use (45).

### 7.5 Splenic complications

Although not part of the GI tract proper, the spleen is an intra-abdominal organ affected by the same vasoconstrictive and thrombotic processes that damage the bowel. Reported splenic pathologies include infarction, hematoma, and atraumatic rupture, which can present with acute abdominal pain and be life-threatening if not recognized promptly.

Cocaine-induced splenic injury was first reported by Novielli and Chambers in 1991, describing vasospasm-induced splenic infarct or hematoma with or without rupture (46). Homler et al. subsequently described atraumatic splenic hematoma without rupture or hemorrhage, indicating a contained bleed (47). The majority of reported cases have an underlying hemoglobinopathy; although the precise mechanism is unclear, cocaine use has been shown to cause up to a 20% transient reduction in splenic volume through vasoconstriction (10). Ballard et al. described combined atraumatic splenic laceration/hemoperitoneum and ileal volvulus after intravenous cocaine use (48). Ribeiro et al. described splenic subcapsular hematoma and spontaneous hemoperitoneum in a 23-year-old cocaine user (49), and Lee Ramos et al. described a similar case of atraumatic splenic laceration and hemoperitoneum in a 23-year-old following intranasal use (50). Karthik and Gnanapandithan described splenic hemorrhage in a chronic cocaine user (51). While most reported cases were managed surgically, Azar et al. described a case of splenic hemorrhage managed conservatively in a hemodynamically stable patient (52), and Khan et al. described a case of splenic hemorrhage and hemoperitoneum managed non-surgically with transcatheter splenic artery embolization (53).

### 7.6 Other complications

#### 7.6.1 Gastric antral vascular ectasia

GAVE, also known as “watermelon stomach, “ is a rare cause of upper GI bleeding, first described in 1953. It manifests as chronic anemia with an endoscopic appearance of linear, erythematous ectatic vessels radiating from the pylorus to the antrum. Kravchenko et al. described a case of upper GI bleeding from GAVE following cocaine use (54).

#### 7.6.2 IBD mimic

Cocaine can induce a state of chronic GI inflammation that mimics inflammatory bowel disease, thought to result from a combination of ischemia, direct mucosal injury, and alterations in the gut microbiome. Proposed mechanisms include increased pro-inflammatory cytokines (IL-18, IL-1β) and chemokines (CCL-2, CCL-7, CXCL-10, CCL-11), with increased activation of the transcription factors NF-κB and CDX-2, affecting mucosal barrier integrity and altering gut microbiota (55). Cocaine has also been associated with an elevated systemic inflammatory state, with decreased basal anti-inflammatory markers (e.g., IL-10) and increased pro-inflammatory cytokines (e.g., TNF-α, IL-1β) (5), and has recently been shown to elicit autophagy involving nitric oxide and glyceraldehyde-3-phosphate dehydrogenase signaling (5).

#### 7.6.3 Cocaine-associated acalculous cholecystitis

A 2024 case report described acute cholecystitis attributed to cocaine use in the absence of gallstones, proposing that cocaine-mediated vasospasm and microvascular thrombosis of the cystic and gallbladder-wall vasculature may produce ischemic cholecystitis analogous to the mechanism underlying cocaine-associated bowel ischemia; the authors noted this to be, to their knowledge, the first such reported case, and that no established treatment protocol yet exists for this presentation (59). This remains an isolated report and should be interpreted as hypothesis-generating rather than established causation.

## 8 Differential diagnosis

Because cocaine-associated GI injury has no single pathognomonic finding, it is best regarded as a diagnosis of exclusion supported by a compatible clinical picture, a temporal link to reported or toxicologically confirmed cocaine use, and exclusion of alternative causes. **Table 3** summarizes features that help distinguish cocaine-associated GI disease from its principal mimics.

**Table 3:** Key differential diagnoses for cocaine-associated gastrointestinal injury.

Condition	Distinguishing features versus cocaine-associated GI injury
Atherosclerotic or embolic mesenteric ischemia	Typically occurs in older patients with known cardiovascular risk factors (atrial fibrillation, atherosclerosis, prior embolic events); CT angiography often shows a discrete occlusive thrombus or embolus at a vessel origin, whereas cocaine-associated ischemia is more often non-occlusive/vasospastic and occurs in younger patients without prior vascular disease (56).
Inflammatory bowel disease (Crohn disease/ulcerative colitis)	IBD typically has a more indolent, relapsing-remitting course, extraintestinal manifestations (uveitis, arthritis, skin findings), and characteristic endoscopic/histologic features (granulomas in Crohn disease, continuous mucosal disease from the rectum in ulcerative colitis); cocaine-associated colitis more often has an acute onset temporally linked to drug use and lacks these chronic hallmark features, though biopsy overlap can occur and is a recognized diagnostic pitfall (55).
Other vasculitides (e.g., polyarteritis nodosa, IgA vasculitis)	Vasculitides typically show additional systemic features (rash, renal involvement, elevated inflammatory markers/autoantibodies) and characteristic vessel-wall inflammation on histology, rather than the bland vasospasm/thrombosis typical of cocaine-associated injury.
Infectious colitis	Usually has a prodrome of fever, preceding diarrhea, or sick contacts, and is confirmed by stool studies/culture; response to antimicrobial therapy also helps distinguish it from ischemic, drug-related injury.
Other vasoconstrictor- or drug-induced colitis (e.g., NSAIDs, decongestants, other sympathomimetics, chemotherapy)	Requires a careful medication and substance-use history; levamisole-adulterated cocaine and cocaethylene (cocaine + alcohol) exposure should specifically be considered, as these confounders can independently contribute to vasculopathy (3, 25, 42, 43).
Perforated peptic ulcer disease unrelated to cocaine (e.g., NSAID- or Helicobacter pylori-associated)	Consider standard risk factors (NSAID/steroid use, H. pylori status, smoking); a disproportionately high rate of prepyloric perforation in a young patient without these risk factors should raise suspicion for cocaine exposure (29).
Hypercoagulable states (e.g., malignancy-associated thrombophilia, inherited thrombophilia, antiphospholipid syndrome)	Consider a personal/family history of venous thromboembolism, known malignancy, or a hypercoagulable panel; mesenteric venous (rather than arterial) thrombosis and a lack of temporal association with drug use favor a primary hypercoagulable state over cocaine-related vasospasm.
Radiation colitis/enteritis	Requires a history of prior pelvic or abdominal radiotherapy; injury typically follows the radiation field and has a delayed, chronic course (telangiectasia, fibrosis, stricture) rather than an acute presentation temporally linked to drug use.

## 9 Diagnostic and initial management algorithm

It is intended as a structured aid to clinical judgment rather than a validated clinical decision rule, given the absence of prospective validation studies in this literature.

## 10 Stratified management strategies

Management should be stratified by severity and anatomic site, informed by the pattern-specific evidence reviewed above.

## 11 Polysubstance use and comorbid vascular disease as confounders

A substantial limitation of the cocaine-GI literature, raised explicitly by the reviewer, is that polysubstance use and pre-existing vascular disease are common in this population but are rarely analyzed as independent variables.

### 11.1 Alcohol co-use and cocaethylene

Concurrent alcohol use is frequent among cocaine users and generates cocaethylene, a metabolite with a longer half-life and greater toxicity than cocaine itself (LD50 60–68.8 mg/kg versus 93.0 mg/kg for cocaine) (42). Early case reports of fatal cocaine-associated intestinal ischemia specifically implicated combined cocaine-alcohol overdose, and the causal contribution of alcohol relative to cocaine alone in these historical cases was not clearly separable (13). In the MASH cohort, concomitant cocaine-and-alcohol users with detectable cocaethylene had a substantially higher odds ratio for liver fibrosis (4.97) than cocaine-only users (1.3 relative to non-users), directly implicating alcohol co-use as an effect modifier of cocaine-related hepatotoxicity rather than a purely incidental exposure (43). A broader systematic review of cardiovascular risk from combined cocaine-alcohol use similarly concluded that combined use carries higher mortality than cocaine alone, while noting that the independent contribution of confounders such as tobacco smoking and pre-existing comorbidity is poorly or inconsistently reported across the underlying studies (60). The same limitation applies directly to the GI literature reviewed here: few reports systematically document or control for concurrent alcohol use, and its independent contribution to the vasospastic and thrombotic mechanisms described above cannot be fully separated from that of cocaine alone.

### 11.2 Tobacco use

Tobacco smoking is highly prevalent among cocaine users and is itself an independent risk factor for atherosclerosis, vasospasm, and impaired mucosal healing (e.g., in peptic ulcer disease). Because tobacco-use status is inconsistently reported in the cocaine-GI case-report literature, its contribution to reported perforation, ulceration, and ischemic events cannot be reliably partitioned from that of cocaine itself; this should be regarded as an important source of residual confounding when interpreting the case-level evidence summarized above.

### 11.3 Levamisole adulteration

Levamisole is a veterinary anthelmintic used to adulterate cocaine and is independently associated with vasculopathy and agranulocytosis; as of July 2009, the US Drug Enforcement Administration identified levamisole in up to 69% of cocaine samples analyzed in the United States (25). Levamisole-induced agranulocytosis increases susceptibility to bowel infection and may compound cocaine’s own vasospastic and thrombotic effects, producing a distinct vasculopathic pattern of ischemic necrosis reported in cocaine users (3, 25). Khan et al. reported a case of recurrent levamisole-induced agranulocytosis complicated by bowel ischemia in a rectal cocaine user, in which recurrent neutropenic episodes closely tracked continued drug exposure (25). Because levamisole content varies unpredictably between cocaine batches and is essentially never confirmed toxicologically in the case-report literature reviewed here, its independent contribution to reported bowel necrosis and enterocolitis — as distinct from cocaine’s intrinsic vasoconstrictive and pro-thrombotic effects — cannot be reliably quantified from existing reports; toxicological confirmation of levamisole exposure should be considered a research priority, as noted in the Stratified Management table and Limitations section below.

### 11.4 Pre-existing vascular disease and hemoglobinopathy

,1A meaningful proportion of reported splenic complications occurred in patients with an underlying hemoglobinopathy (e.g., sickle cell trait/disease), which independently predisposes to splenic infarction; cocaine-induced splenic vasoconstriction may act as an additional precipitant rather than a sole cause in these cases (10, 46, 47). Similarly, older patients with reported cocaine-associated bowel ischemia may have coexisting atherosclerotic disease that contributes to, rather than being fully distinguished from, cocaine-related vasospasm — reinforcing the differential-diagnosis considerations in **Table 3**.

### 11.5 Implications for interpretation

Because case reports rarely adjust for these co-exposures, the attribution of GI injury to cocaine alone in much of this literature should be understood as a plausible, temporally supported association rather than a fully isolated causal link. Future comparative studies should systematically document and, where possible, statistically adjust for alcohol, tobacco, and other co-exposures, and should specifically test for cocaethylene and levamisole exposure using toxicological confirmation rather than self-report alone.

## 12 Summary table: complications, mechanisms, and management

**Table 4** consolidates the complications reviewed above with their proposed mechanisms and first-line management.

**Table 4:** Complications of cocaine-associated gastrointestinal injury, proposed mechanisms, and first-line management.

Complication	Proposed mechanism(s)	First-line management
Acute/chronic mesenteric ischemia	Splanchnic vasospasm; endothelin-1–mediated vasoconstriction; arterial thrombosis	CT angiography; supportive care if non-occlusive and stable; surgical/endovascular intervention for occlusive or transmural disease
Ischemic/hemorrhagic colitis	Vasospasm of watershed colonic vessels; mucosal hypoxia-reperfusion injury	Colonoscopy for diagnosis; conservative management for mucosal disease; resection for transmural necrosis or perforation
Cocaine-induced enterocolitis	Chronic ischemia and mucosal inflammation	Supportive care; cessation of use; surgery reserved for peritonitis
Acute esophageal necrosis (“black esophagus”)	Distal esophageal ischemia + reflux-related mucosal injury	NPO, PPI/sucralfate; surgery for perforation
Gastroduodenal/gastric/duodenal perforation	Vasoconstrictive ischemia + direct mucosal injury + increased intragastric pressure (smoking)	Emergent surgical repair; antiulcer procedure preferred over simple omental patch
Small-/large-bowel perforation	Transmural ischemic necrosis with rupture	Surgical resection of affected segment
Fibrotic strictures/obstruction	Chronic ischemia-driven fibrosis	Endoscopic dilation or surgical reconstruction (e.g., Roux-en-Y) depending on site/severity
Acute pancreatitis	Vasoconstriction; thrombotic microangiopathy	Standard supportive pancreatitis care; monitor for necrosis/multi-organ failure
Hepatotoxicity/acute hepatitis/liver failure	Hepatotoxic metabolites (norcocaine, cocaethylene); oxidative stress; mitochondrial injury	Supportive care; cessation of cocaine and alcohol; monitor for TTP/liver failure
Cocaine-associated acalculous cholecystitis	Proposed cystic/gallbladder-wall vasospasm and microthrombosis (single report)	Standard cholecystitis management; no established cocaine-specific protocol
Splenic infarction/hematoma/rupture	Splenic vasoconstriction (up to 20% transient volume reduction); may be compounded by hemoglobinopathy	Conservative management or transcatheter embolization if stable; splenectomy/splenorrhaphy if unstable
GAVE (“watermelon stomach”)	Chronic mucosal vascular ectasia (mechanism in cocaine-associated cases not fully established)	Endoscopic argon plasma coagulation; iron replacement as needed
IBD-mimicking colitis	Cytokine-mediated mucosal barrier disruption and dysbiosis	Distinguish from true IBD (**Table 3**); supportive care and cessation

## 13 Quality of the evidence and critical appraisal

Readers should interpret the foregoing synthesis with explicit awareness of the underlying evidence hierarchy, which is heavily skewed toward the lowest levels of clinical evidence:

- Predominance of case reports and small case series. The large majority of citations in this review — including virtually all entries describing acute esophageal necrosis, gastric antral vascular ectasia, splenic complications, fibrotic/obstructive strictures, cholecystitis, and hepatobiliary/pancreatic injury — are single case reports or small case series (typically fewer than 10 patients). Such reports are inherently subject to selection and publication bias (unusual or dramatic presentations are disproportionately likely to be published), cannot establish true incidence or absolute risk, and provide only a weak basis for causal inference.
- Comparative studies remain rare. Three studies identified in this review provide comparative or aggregated quantification of risk: Elramah et al. (16) and Niazi et al. (17), both retrospective cohort/case-control analyses confined to ischemic colitis, and Farooq et al. (57), a systematic review of 53 studies (69 patients) examining outcomes by route of cocaine use across the wider intestinal-ischemia literature. These are the most robust quantitative data in this literature, but even these are retrospective, single- or few-center (or aggregate case-level), and subject to residual confounding (e.g., unmeasured polysubstance use, delayed presentation related to reluctance to disclose drug use, or differential access to care).
- Causal plausibility versus proof of causation. The mechanistic literature (vasospasm, endothelin-1–mediated vasoconstriction, platelet activation, a pro-thrombotic coagulation profile, and direct cytotoxic/oxidative injury) provides strong biological plausibility for a causal relationship between cocaine and GI ischemic/ulcerative injury. However, most clinical reports rely on temporal association (recent reported cocaine use plus a compatible clinical/imaging/histologic picture) rather than controlled comparison, and cannot fully exclude confounding by co-ingested substances (notably alcohol, forming cocaethylene, and levamisole, a common adulterant independently associated with vasculopathy and agranulocytosis) or by underlying vascular risk factors, as detailed in the Confounders section above.
- Heterogeneity and lack of standardized case definitions. Diagnostic criteria, imaging/endoscopic thresholds, and terminology (e.g., “cocaine enteropathy,” “cocaine colitis, “cocaine gut”) are not standardized across reports, limiting comparability and precluding meaningful pooled quantitative synthesis.
- Reporting and language bias. Restriction to English-language reports (as in most narrative reviews of this literature, including the present one) may omit relevant non-English case series, and indexing gaps mean that some published cases may not be retrievable through the search strategy described above.

Taken together, this body of evidence supports cocaine use as a biologically plausible and clinically important — but incompletely quantified — cause of severe GI morbidity and mortality. The strength of individual recommendations in this review should be understood as resting on expert clinical experience and mechanistic plausibility for most complications, with only the ischemic-colitis and route-of-use literature offering comparative, quantitative support.

## 14 Limitations of this review

- This is a narrative, not a systematic, review: no PRISMA flow diagram with adjudicated database counts, dual independent screening, formal risk-of-bias/quality appraisal instrument (e.g., the CARE checklist for case reports, or Newcastle–Ottawa Scale for cohort/case-control studies), or GRADE certainty rating was applied, for the reasons discussed above; **Table 1** documents the actual single-reviewer search and selection process used.
- The underlying literature is dominated by case reports and small series, most published before 2015; this revision incorporates several 2021–2025 reports and two systematic reviews, but the field continues to rely disproportionately on older anecdotal data, and true contemporary incidence remains unknown.
- Because inclusion required English-language material (or a full translation), some relevant non-English literature may have been missed.
- No independent, blinded re-abstraction of primary-source clinical data (e.g., age, sex, or route of use) was performed for every cited report; where such details were not clearly reported in the source publication, they are marked “NR” in this manuscript rather than inferred.
- Publication bias likely favors severe, unusual, or diagnostically instructive presentations, which may overstate the perceived severity of typical cocaine-associated GI disease relative to its true population-level burden.
- As detailed in the Confounders section, polysubstance use (alcohol, tobacco, levamisole) and pre-existing vascular disease are inconsistently reported across the underlying case literature and could not be systematically adjusted for in this review.
- The proposed diagnostic algorithm and stratified management table (**Table 5**) are original syntheses by the authors intended to aid clinical reasoning; they have not been prospectively validated and should not be applied as a substitute for individualized clinical judgment.

**Table 5:** Stratified management strategies by severity and presentation.

Severity/presentation	Recommended initial approach
Mild, self-limited mucosal ischemia/enteritis (no peritonism, hemodynamically stable)	Supportive care: bowel rest, intravenous fluids, analgesia, cessation counseling, and close clinical monitoring with serial abdominal examination; repeat imaging or endoscopy if symptoms fail to improve within 24–48 hours (2, 19).
Ischemic colitis without transmural necrosis or perforation	Conservative management (bowel rest, fluids, broad-spectrum antibiotics if concern for bacterial translocation) is often sufficient, but close monitoring is essential given the disproportionately high mortality reported in cocaine-associated cohorts compared with other causes of ischemic colitis (16, 17).
Perforation (gastric, duodenal, small-, or large-bowel) or transmural infarction	Emergent surgical consultation for resection/repair (primary repair, omental patch, or segmental resection as indicated); antiulcer surgical technique rather than simple omental patch alone may reduce recurrence at gastric perforation sites (32).
Acute esophageal necrosis (“black esophagus”)	Strict nil-by-mouth, proton-pump inhibitor and/or sucralfate, and monitoring for perforation (occurs in ~7% of cases); surgery reserved for perforation or failure of conservative management (21).
Massive pan-gastrointestinal ischemia/bowel gangrene	Immediate laparotomy with resection of necrotic bowel and aggressive resuscitation; recognize that ongoing vasoconstriction may continue to threaten remaining bowel postoperatively, and consider vasodilator therapy (e.g., papaverine) as an adjunct where no perforation is present (24, 56).
Splenic hematoma/rupture	Hemodynamically stable, contained injuries may be managed conservatively or with transcatheter splenic artery embolization; unstable patients or free rupture require surgical management (splenectomy or splenorrhaphy) (52, 53).
Levamisole-adulterated cocaine exposure with agranulocytosis	Cocaine cessation, monitoring of absolute neutrophil count, and heightened suspicion for infectious and ischemic bowel complications; consider toxicological confirmation of levamisole exposure where available (3, 25).
All presentations	Substance-use cessation counseling and, where appropriate, referral to addiction medicine services should be integrated into discharge planning, given the likelihood of recurrence with continued use.

Recommendations are derived from case-level and small comparative studies rather than randomized trials, and should be applied with clinical judgment.

Future work should prioritize multicenter or registry-based cohort studies (analogous to the Elramah, Niazi, and Farooq studies) across the full spectrum of cocaine-associated GI disease, standardized case definitions, systematic documentation of co-exposures (alcohol, tobacco, levamisole), and, where feasible, biomarker or toxicological confirmation of adulterant exposure, to allow more rigorous quantification of risk and to support evidence-graded clinical guidance.

## 15 Conclusion

Cocaine is a potent vasoactive and cytotoxic agent capable of inducing a broad spectrum of gastrointestinal injuries through mechanisms involving intense vasospasm, endothelial dysfunction, thrombosis, oxidative stress, and direct mucosal toxicity. Reported clinical manifestations range from self-limited ischemic colitis to catastrophic mesenteric ischemia, bowel infarction, and perforation. Extraintestinal involvement — including hepatotoxicity, pancreatitis, splenic infarction, and, rarely, acalculous cholecystitis — further underscores the systemic impact of cocaine use.

As detailed above, this evidence base rests overwhelmingly on case reports and small series, with only three comparative or aggregated studies (two limited to ischemic colitis, one examining route of use across intestinal ischemia broadly) providing quantitative risk data, and with polysubstance use and comorbid vascular disease acting as incompletely characterized confounders throughout. Clinicians should therefore maintain a high index of suspicion for cocaine-related gastrointestinal pathology — particularly in younger patients presenting with unexplained abdominal pain or ischemic findings — use the differential-diagnosis framework and algorithm proposed here to avoid diagnostic delay, and recognize that true incidence, absolute risk, and the independent contribution of adulterants such as levamisole and of co-exposures such as alcohol and tobacco remain incompletely defined. Early recognition, prompt diagnostic imaging, and timely, severity-stratified intervention remain critical to improving outcomes.

Future studies should focus on elucidating the molecular pathways underlying cocaine-induced gastrointestinal injury, identifying predictive biomarkers, conducting multicenter or registry-based comparative studies to quantify true incidence and risk while adjusting for polysubstance confounders, prospectively validating diagnostic algorithms such as the one proposed here, and establishing evidence-graded management protocols to mitigate the morbidity and mortality associated with this under-recognized clinical entity.
